# Development of a Novel Energy Saving and Environmentally Friendly Starch via a Graft Copolymerization Strategy for Efficient Warp Sizing and Easy Removal

**DOI:** 10.3390/polym16020182

**Published:** 2024-01-08

**Authors:** Yuhan Zhu, Fei Guo, Jing Li, Zhen Wang, Zihui Liang, Changhai Yi

**Affiliations:** National Local Joint Laboratory for Advanced Textile Processing and Clean Production, State Key Laboratory of New Textile Materials and Advanced Processing Technologies, Wuhan Textile University, Wuhan 430073, China

**Keywords:** starch, copolymerization, warp sizing, desizing, energy-saving and environmental-friendly

## Abstract

Warp sizing is a key process in textile production. However, before the yarn/fabric finishing, such as dyeing, the paste adhering to the warp must be eliminated to ensure optimal dyeing properties and the flexibility of the fabric. Therefore, the sizing will often consume a lot of energy and produce a lot of industrial wastewater, which will cause serious harm to the environment. In this study, we have developed an energy saving and environmentally friendly starch-based slurry by modifying natural starch with acrylamide. The paste has excellent viscosity stability and fiber adhesion, and exhibits excellent performance during warp sizing. In addition, the slurry has good water solubility at 60–70 °C, so it is easy to desize at low temperatures. Because of this, the sizing of the warp can be deslimed directly from the yarn during subsequent washing processes. This work can not only reduce some costs for the textile industry, but also achieve the purpose of energy conservation and emission reduction.

## 1. Introduction

It is well known that the warp yarn will be repeatedly subjected to a series of mechanical forces during the weaving process, so that the phenomenon of frequent breakage of the warp will result in low weaving efficiency [1,2], Therefore, warp sizing is a critical process in textile production, which forms a film on the surface of the yarn to improve the mechanical properties of the warp, protecting the yarn so it is not easy to break. As a renewable natural polymer compound in nature, starch has become an important textile sizing material due to its availability, low price and biodegradability [3,4]. However, starch molecular chains are composed of cyclic glucose residue groups, which leads to poor flexibility of macromolecules, resulting in the sizing films exhibiting brittle and hard properties [5,6]. These drawbacks of natural starch seriously limit its application in textile warp sizing. Therefore, developing a new starch-based slurry that can meet the environmental requirements of warp sizing in the textile industry is an important issue and needs to be addressed [7].

Considerable efforts to improve the sizing properties of starch-based slurry to meet the requirements of warp sizing have mainly focused on oxidation [8], hydrolysis [9], graft copolymerization [10,11] and crosslinking [12]. Among the various strategies, graft copolymerization is promising approach for improving the sizing properties of starch and expanding its range of applications [13]. Meshram et al. proposed styrene (ST), butyl acrylate (BA) and methyl methacrylate (MMA) as monomers and ferrous ammonium sulfate–hydrogen peroxide initiated reaction, the optimum monomer concentration and initiator concentration were investigated. ST/BA and ST/MMA grafted starch showed superior tensile strength for cotton sizing [14]. Zhu et al. reported that acid starch (ATS) and 2-acryloxyethyl trimethyl ammonium chloride (ATAC) initiated grafting copolymerization in the ferrous ion redox system, and found that grafted starch with the best grafting percentage of 7.5% significantly improved the adhesion properties of starch with cotton fiber and polyester fiber [15]. Zha et al. studied the grafting parameters of starch and polyacrylic acid (PAA) under different conditions, using ammonium cerium nitrate as initiator, and observed that the abrasion resistance of the sizing yarns was effectively improved, while maintaining a better desizing effect [16]. Zhu et al. employed a series of maleate starches with different degree of substitution (DS) values prepared by treating maleates with maleic anhydride, and the starch films had better elongation and breaking strengths and greater bending resistance after employing low levels of maleation and sulfosuccinic acidification [17]. Djordjevic et al. successfully found that azobisisobutyronitrile triggered a graft polymerization with starch hydrolyzed by hydrochloric acid, and the grafted starch was used for sizing of cotton yarns with a more homogeneous distribution of the slurry in the fiber [18].

Previous researchers have made important contributions to the modification of natural starch-based slurry, which has great significance in promoting the development of warp sizing [19]. Most of the research focuses on the type of initiator, the concentration of monomer, the type of monomer and the conditions of graft copolymerization reaction to improve the grafting percentage, grafting efficiency and sizing performance. However, the biodegradability and desizing properties of starch-based slurry are very important for industrial production. As mentioned above, the slurry is used to improve the warp weave. When the fabric is finished, a desizing process is also needed to remove it from the surface of the warp. The common desizing methods are acid hydrolysis and enzyme hydrolysis, depending on the composition of the slurry [20]. During the process, a large amount of energy is often consumed and a large amount of industrial wastes are generated, which causes serious harm to the environment [21,22]. In this study, we developed an energy saving and environmentally friendly starch-based slurry (abbreviated to St-AM) via modifying natural starch (St) with acrylamide (AM), which significantly enhances viscosity stability, coagulation resistance and fiber adhesion of the St-AM slurry with excellent warp sizing performance. Moreover, the St-AM slurry exhibits excellent water solubility at 60–70 °C, so the slurry has excellent sizing performance and easy desizing at low temperatures. Because of this, the warp can be receded directly after sizing during subsequent washing processes, thus reducing the traditional desizing process. Overall, it will not only reduce some costs for the textile industry, but also achieve the purpose of energy conservation and emission reduction.

## 2. Materials and Methods

### 2.1. Materials

Pure cotton roving (672 tex) was used to determine fiber adhesion. Pure cotton yarn (29 tex) was supplied by Hubei Deyongsheng Textile Company Limited (Shishou, China). Natural potato starch (industrial grade) and acrylamide were purchased from Aladdin Industrial Company (Shanghai, China). Other reagents such as ammonium persulfate (APS), methanol, ethanol, glacial acetic acid and N, N-dimethylformamide were provided by Sinopharm Chemical Reagent Co. (Shanghai, China).

### 2.2. Preparation of St-g-PAM

Prior to graft copolymerization, potato starch was subjected to acid digestion to provide a control sample (ACS) for subsequent work. An amount of acid-dissolved starch was weighed and configured into a 4.3% starch suspension. This suspension was transferred into a four-necked flask equipped with a thermometer, condenser tube, dropping funnel and nitrogen conduit, and pasted at 65 °C for 30 min. Subsequently, when cooled down to 50 °C, the initiator, ammonium persulfate (for 4% of the mass of the dry starch) was added slowly dropwise to pre-react with the starch macromolecules for 20 min. A certain amount of acrylamide solution (configured as 10% aqueous) was then added slowly dropwise by dropping the starch with the use of a dropping funnel, so that the dropwise addition was completed within 30–40 min and then the mixture was stirred for 3 h. Finally, 50 mL methanol was added to the mixture to terminate the polymerization reaction, then the mixture was precipitated, washed with ethanol 3–4 times, and filtered for collection. The grafted samples were marked as St-g-PAM 1 (starch to acrylamide mass ratio 2:1), St-g-PAM 2 (starch to acrylamide mass ratio 1:1), St-g-PAM 3 (starch to acrylamide mass ratio 1:1.5), and St-g-PAM 4 (starch to acrylamide mass ratio 1:2). The crude product obtained was wrapped in filter paper and extracted at reflux in a mixed solvent of glacial acetic acid (AC) and N,N-dimethylformamide (1:1, *v*/*v*) for three siphon cycles. The product was then washed three times with ethanol, and the washed product was dried in a vacuum drying oven for 12 h to remove the homopolymer in the reaction.

### 2.3. Characterization

Monomer conversion refers to the proportion of monomers converted to polymers as a percentage of incorporated monomers [23]. The grafting percentage refers to the mass of copolymer grafted onto the molecular chain of starch as a proportion of the mass of starch, and the grafting efficiency refers to the mass of grafted copolymer as a proportion of the total mass of the initially invested monomer and starch [24]. They are calculated according to Equations (1)–(3).

(1) $$\mathrm{Monomer}\;\mathrm{conversion}\;\mathrm{ratio}\left(\%\right)=\frac{m_{1}{-m}_{2}}{m_{1}}\times 100$$

(2) $$\mathrm{Grafting}\;\mathrm{percentage}\;\left(\%\right)=\frac{m_{3}-\;m_{4}}{m_{4}}\times 100$$

(3) $$\mathrm{Grafting}\;\mathrm{efficiency}\;\left(\%\right)=\frac{m_{3}}{{m_{1}+m}_{4}}\;\times \;100$$

where m_1_, m_2_, m_3_ and m_4_ are the masses of monomer used, residual monomer, graft copolymer onto the molecular chain of starch and starch, respectively. In the other words, m_1_ is the mass of acrylamide weighed in the grafting reaction, m_4_ is the mass of starch added in the grafting reaction, m_3_ is the mass of grafted starch produced after the grafting reaction, and m_2_ is the mass of acrylamide not involved in the grafting reaction, which is equal to the value of m_1_ − (m_3_ − m_4_).

The viscosity of the slurry was measured using a Wu-type viscometer [25]. Firstly, the starch aqueous suspension with a concentration of 6% was configured, and then heated at 95 °C for 1 h to prepare a starch paste. The viscosity was recorded every 30 min for five consecutive times. The viscosity stability was calculated according to Equation (4).

(4) $$\mathrm{Vs}\;\left(\%\right)=(1-\frac{\eta_{\max}\;-\;\eta_{\min}}{\eta_{t60}})\times 100$$

where η_max_ and η_min_ denote the maximum and minimum viscosity recorded over a period of time, respectively, and η_t60_ refers to the viscosity recorded at 60 min. The adhesion of pulp to fibers was investigated using the FZ/T 15001-2017, and the adhesion properties of pulp to fibers were determined according to the literature [26]. Tensile tests were carried out on an Instron universal material testing machine at a speed of 50 mm/min and an initial clamping distance of 100 mm, and each specimen was measured 20 times to take the average value.

FTIR spectra were obtained using a Perkin-Elmer spectrometer with a test wavelength range of 3000 to 500 cm^−1^. XRD was tested using an Empyrean-type X-ray diffractometer with a scanning range of 10–80° and a scanning time of 3 min. A scanning electron microscope (SEM) (JSM-7001F, JEOL) was used to analyze the microstructure and chemical composition of the samples. Thermogravimetry was measured using an NETZSCH TG209F1-Nevio-Thermogravimetric Analyzer (Selb, Germany), with a temperature range of 30–650 °C and a temperature increase rate of 10 °C/min. Biochemical oxygen demand (BOD_5_) was determined according to the Italian standard method IRSA-CNR 29/2003-5120-B2. A volume of the eluate to be tested was placed in a Winkler bottle (volume = 300 mL). The bottle was then filled with dilution water saturated in oxygen and containing bacterial inoculum and the nutrients required for biological growth. The bottle was stored in the dark at a temperature of 20 °C for 5 d. The oxygen concentration in the bottle before and after 5 d of incubation was measured by adding to the solution manganese sulfate and potassium iodide in sodium azide and titrating the residue iodine with sodium thiosulphate. The transmittance was determined using a UV-101 spectrometer with distilled water as the reference sample (the transmittance was 100%) at wavelengths ranging from 200 nm to 800 nm. The water sample was extracted to measure chemical oxygen demand (COD_cr_) with a HACH COD reactor, which was expressed as COD_Cr_ (potassium dichromate as oxidant).

## 3. Results and Discussion

### 3.1. Grafting Process and Mechanism

**Figure 1:**
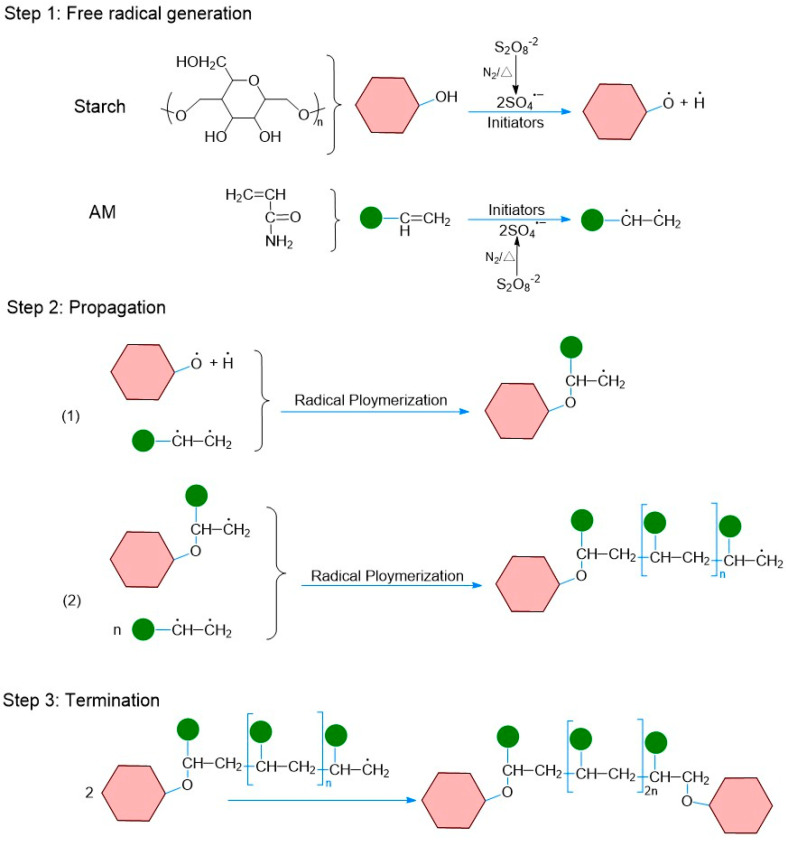
Synthesis process of grafted starch slurry.

Figure 1 depicts the chemical mechanism of the graft copolymerization reaction between starch and acrylamide. The water-soluble initiator APS dissociates at a suitable temperature to produce a pair of initiating radicals (SO_4_). A portion of these initiating radicals diffuse in water, taking hydrogen atoms from the hydroxyl groups of polysaccharides and from the alkyl groups of monomers to create active site initiation reactions in the starch backbone (step 1). The subsequent chain growth stage (step 2) involves two reactions in which a single unit of acrylamide monomer is added to the starch backbone (1), and then the length of the grafted chain is increased by attaching more acrylamide monomers to the starch backbone (2). The chain termination stage (step 3), in which two growing chains are coupled together, terminates the growth of the latter chain. The grafted starch samples were prepared in aqueous medium using starch and acrylamide as raw materials and the entire synthesis process is shown in Figure S1.

### 3.2. Characterization of St-g-PAM

**Figure 2:**
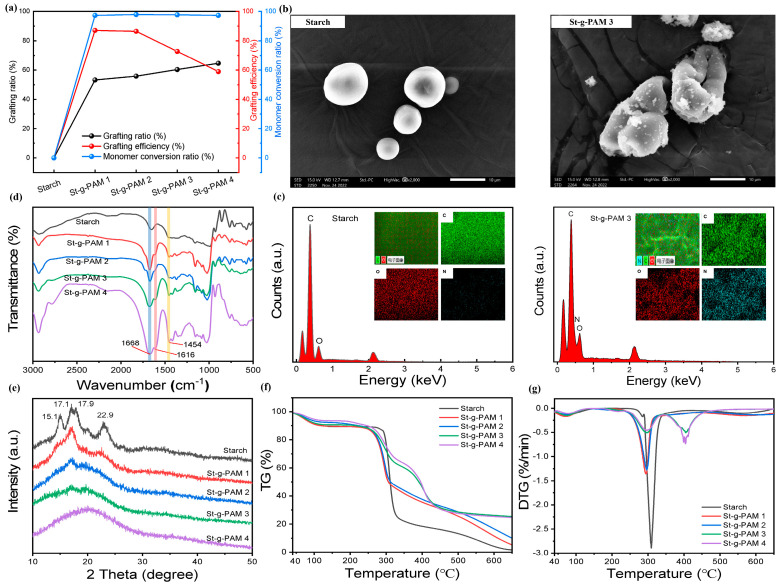
(**a**) Grafting parameters of starch and St-g-PAM with different mass ratios. (**b**) SEM images and (**c**) EDS spectrogram of starch and St-g-PAM 3. (**d**) FT-IR spectra and (**e**) XRD image of starch and St-g-PAM with different mass ratios. (**f**) TGA and (**g**) DTG curves of starch and St-g-PAM with different mass ratios.

As shown in Figure 2a, we evaluated the effect of different mass ratios of St-g-PAM on the grafting percentage and grafting efficiency. The results showed that with the increase of AM monomer dosage, more monomers were attached to the free radicals on the starch backbone in the system, and thus the grafting percentage increased from 0 to 64.6%. On the other hand, the grafting efficiency of St-g-PAM showed a decreasing tendency due to the increase of the dosage of AM monomers, and as the grafting reaction continued, the active sites on the starch granules were continuously occupied, and thus there were not enough sites for the introduction of new PAM branching. In addition, the monomer-to-polymer conversions were all over 97%, indicating that most of the monomers had already undergone polymerization reactions to polymers. The detailed grafting parameters are listed in Table S1.

The particle morphology of starch before and after modification was investigated using scanning electron microscopy (SEM), as shown in Figure 2b and Figure S2. The surface of the granules of natural starch was smooth and had an elliptical structure, and the structure of St-g-PAM granules changed significantly after graft copolymerization. Compared with the pristine starch, the grafted starch particles were more irregular, the surface was rough, and all of them showed different degrees of depression, and these phenomena indicated that the graft modification occurred not only on the surface of the particles but also inside the particles. The EDS technique has become an important method for determining the chemical composition of polymeric materials [27]. The energy dispersive X-ray (EDX) spectroscopy confirms the main elements of the starch and St-g-PAM 3, as shown in Figure 2c. The pristine starch exhibited C and O elements, which corresponds to the composition of natural starch. In contrast, the presence of a new N element on the surface of St-g-PAM 3 indicates that the PAM side chain was successfully grafted onto the starch. In addition, the corresponding mapping of the starch and St-g-PAM 3 (the insets of Figure 2c) further confirmed the findings. To ensure the robustness of these observations, similar analyses were conducted on the St-g-PAM 1, St-g-PAM 2, and St-g-PAM 4, as shown in Figure S3. As the amount of PAM added increased, the amount of elemental N increased. The elemental composition of N indicates that the PAM side chain was successfully grafted onto the starch.

The FT-IR spectra of starch and St-g-PAM with different raw material ratios are shown in Figure 2d. In addition to the characteristic peaks of the starch itself, some new characteristic peaks appeared in the spectrum of St-g-PAM. There are sharp peaks at 1668 cm^−1^ and 1616 cm^−1^ that correspond to C=O stretching and N-H bending of the -CONH_2_ group in acrylamide, respectively [28]. There is also an additional peak at 1454 cm^−1^ which is C-N bond stretching [29]. Thus, these results indicate that the acrylamide unit has been successfully grafted onto the starch backbone. Figure 2e shows the XRD patterns of the original starch and St-g-PAM. For the original starch, there are four obvious diffraction peaks at 2θ = 15.1°, 17.1°, 17.9° and 22.9°, but the intensity of the crystalline peaks of the modified grafted starch is obviously reduced. This shows that graft copolymerization increases the difficulty of neatly aligning the starch chain [30]. Thus, as the amount of monomer input increases, the grafting percentage gradually increases and the more PAM branches are introduced into the starch chain, the more significantly crystallinity decreases.

The TGA and DTG curves of starch and St-g-PAM samples at 30–650 °C under nitrogen are shown in Figure 2f,g. Starch involves two distinct mass loss regions, with an initial moisture mass loss at around 100 °C, which may be due to the separation of free water molecules present in starch. Moreover, there is a second mass loss zone between 250–330 °C, which is due to the breakdown of the glycosidic bonds in the starch chains. Finally, the grafted starch also has an additional region of mass loss (350–450 °C), which is due to the decomposition of the PAM grafted side chain portion of the starch chain, further indicating that the grafting of starch was successful [31].

### 3.3. Slurry and Film Properties

**Figure 3:**
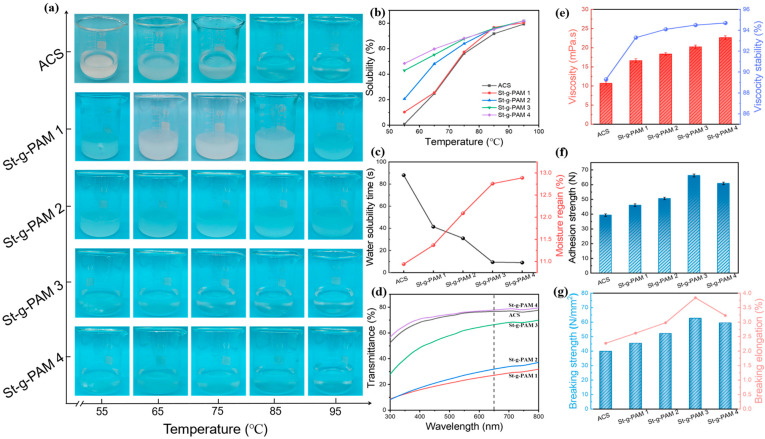
(**a**) Optical images depicting water solubility at different temperatures. (**b**) water solubility, (**c**) solubility time and moisture regain, (**d**) UV-visible transmittance of slurry ACS and St-g-PAM with different raw material ratios. (**e**) viscosity and stability, (**f**) adhesion, (**g**) breaking strength and elongation of slurry films ACS and St-g-PAM with different raw material ratios.

Hydration capacity is an important physicochemical property of slurry. Water solubility will lead to good permeability, flow and wettability, and good swelling will increase the adhesion of starch and the stability of slurry. Generally, the hydrophilicity of starch can be reflected by the degree of swelling of starch in water [32]. The water-soluble optical images of ACS and St-g-PAM samples at different temperatures are shown in Figure 3a. It is clearly observed that the pristine starch cannot be dissolved in the 55 °C water and is deposited in the bottom of the beaker. When the water is heated to 95 °C, starch is completely dissolved due to the water molecules connecting with the hydroxyl groups on the starch molecules through hydrogen bonding, resulting in the swelling and dissolution of the granules [33]. Compared with pristine starch, the solubility of St-g-PAM in water was significantly enhanced at the same temperature. In particular, St-g-PAM 3 and St-g-PAM 4 can form a uniform transparent solution in water at 55 °C due to the increase of amide groups, which are hydrophilic. The corresponding solubility is shown in Figure 3b. At 55 °C, the ACS is in a stratified state and the water solubility tends to 0. However, the solubility of the grafted starch is significantly increased. In addition, as the temperature rises, the solubility of ACS and St-g-PAM samples increased significantly, eventually reaching over 80%. Therefore the stronger the water solubility of the grafted starch, the more easily the particles are dispersed in water. Details of the corresponding water solubility are shown in Table S2. In addition, the swelling of ACS and St-g-PAM samples at different temperatures are shown in Figure S4. It is clearly observed that the swelling of all modified starches is better than that of the original starch (ACS) when the water temperature is below 85 °C. Swelling parameters in detail are listed in Table S3. Figure 3c shows the water solubilization time and moisture regain of ACS and St-g-PAM films. The branched chains grafted onto the starch chains greatly shortened the water solubilization time of the St-g-PAM films due to their hydrophilic properties and they were able to absorb moisture from the air, resulting in a significantly higher moisture regain of the St-g-PAM films than that of the ACS films. The transmittance of the slurry reflects the antiagglomeration performance of the slurry and directly reflects its dispersion stability. The solution of starch and modified starch dissolved in water at 90 °C was tested for transmittance, and the results are shown in Figure 3d. The transmittance increases gradually with the increase of PAM branches, and the transmittance of St-g-PAM 3 is comparable to that of ACS. The more hydrophilic branches are introduced, the more hydrophilic St-g-PAM is, and the more fully particles are dissolved. Optical photographs of the corresponding slurry films are shown in Figure S5.

Viscosity and stability are important parameters for evaluating the performance of slurry. Graft polymerization has a significant effect on starch paste viscosity, as shown in Figure 3e, which indicates that the slurry viscosity is increasing with the increase of grafting percentage and the viscosity and stability of the modified starch paste has been greatly improved [34]. This may be attributed to the increase in molecular weight and the hydrophilicity of the grafted side chains, which improves the force between starch and water molecules, and increases the resistance to flow of the paste. Undoubtedly, the St-g-PAM slurry, with a stability greater than 90%, is fully capable of meeting the requirements for stability during the sizing process, thus ensuring the stability of sizing. The adhesion of the paste to the fibers helps to bond the fibers together to improve the strength and abrasion resistance of the sizing yarns, and to reduce the fluffiness or hairiness of the yarns, which ultimately improves the weavability of the sizing yarns [35]. The bonding properties of St-g-PAM with cotton fibers in different mass ratios were evaluated, as shown in Figure 3f. The results showed that the adhesion of St-g-PAM to cotton fibers was higher than that of ACS in all cases. With the increase of the ratio, the adhesion of cotton fibers increased from 39.3 N to 66.22 N and then decreased to 60.84 N, which indicated that the introduction of the PAM branch played a crucial role in improving the adhesion between starch and cotton fibers. When the mass ratio of starch and monomer was 1:2, the adhesion decreased slightly, indicating that the viscosity of the slurry was too high, which was unfavorable for yarn impregnation and draping. The cotton roving was impregnated in the slurry, then the slurry adhered to the surface of the roving, and finally a layer of adhesive was formed between the fibers. Figure 3g shows the effect of PAM branching on the tensile properties of the pulp films. The results show that the starch films prepared by graft copolymerization have superior elongation at break as well as tensile strength compared to the control starch ACS. From the figure, it can be seen that St-g-PAM 3 film has the highest elongation (3.84%) and the highest breaking strength (62.62 N·mm^−2^), while the ACS film has the lowest elongation (2.27%) and the lowest breaking strength (39.86 N·mm^−2^). This indicates that St-g-PAM films have superior properties compared to ACS films and are more suitable for warp sizing. The tensile strain of slurry films ACS and St-g-PAM with different raw material ratios are shown in Figure S6.

### 3.4. Sizing Yarn Morphology and Properties

**Figure 4:**
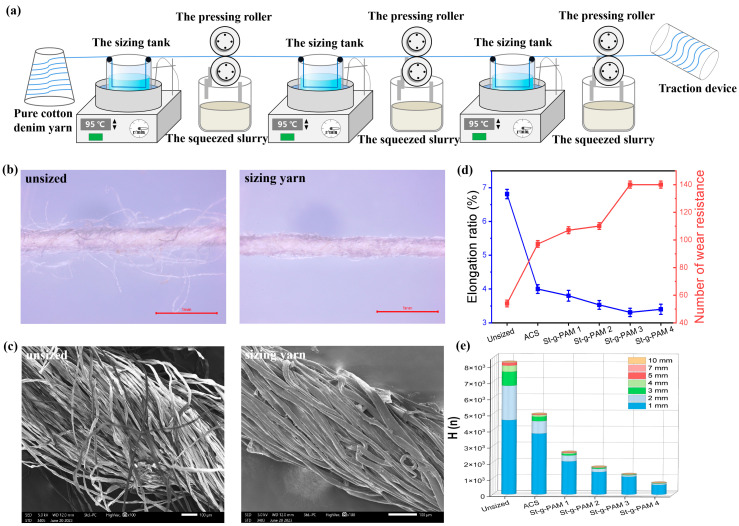
(**a**) Schematic diagram of sizing line. (**b**) Optical microscope images and (**c**) SEM images of unsized and sizing yarn. (**d**) Breaking elongation and abrasion resistance and (**e**) hair feathers of sizing yarns ACS and St-g-PAM with different raw material ratios.

In order to better evaluate the warp sizing performance of modified starch, we established a simple laboratory warp sizing device in a simulated real factory, as shown in Figure 4a. First, cotton denim yarns were sized through the sizing tank, then the residual sizing solution on the yarns was extruded using a squeezing roller, and finally the yarns were dried around the cylinder. The slurry concentration was 6%, the sizing temperature was 95 °C, the sizing speed was 30 m/min, and the drying temperature was controlled at 50 °C. The specific process parameters for pulping in the slurry tank are shown in Table S4. Optical microscope photographs and SEM images of the raw yarns and sizing yarns are shown in Figure 4b,c. It can be seen that the fibers of the raw yarn are loose and there is a lot of hairiness on the surface. After sizing, the slurry forms a film on the surface of the yarn, which makes the fibers stick together, thus reducing the hairiness of the surface of the yarn. Cross-sectional SEM images of the raw yarn and sizing yarn are shown in Figure S7, where it can be observed more intuitively that the fibers in the raw yarn are independent of each other, and the fibers in the sizing yarn are clustered together with smaller inter-fiber gaps. This shows that St-g-PAM is not only on the surface of the yarn but also penetrates into the yarn, which enhances the cohesion between the fibers and the ability to resist external forces, thus protecting the yarn and making it weavable.

The breaking elongation and abrasion resistance of sizing yarns before and after sizing are shown in Figure 4d. Compared with the pristine yarns, the warp yarn after sizing had higher wear resistance and lower elongation at break, which can be attributed to the superior adhesion of St-g-PAM slurry to cotton yarn, resulting in a more effective attachment of the slurry film onto the surface of cotton yarn. Consequently, this protective mechanism contributes to an improved wear resistance of cotton yarn. In addition, the breaking strength and moisture regain of the warp yarn after sizing was obviously improved, as shown in Figure S8. After sizing, the harmful hairiness on the surface of the warp was significantly reduced by more than 3 mm, and the moisture regain was consistent with the results of the sizing film, as shown in Figure 4e. The introduction of a hydrophilic PAM branch chain significantly enhanced the hydrophilicity and water dispersion of starch, thereby increasing the toughness of the starch gel layer while reducing break. During the sizing process, the slurry soaked into the interior of the yarn, which enhanced the holding force of the single fibers within the yarn and improved the strength of the yarn.

### 3.5. Desizing Properties

**Figure 5:**
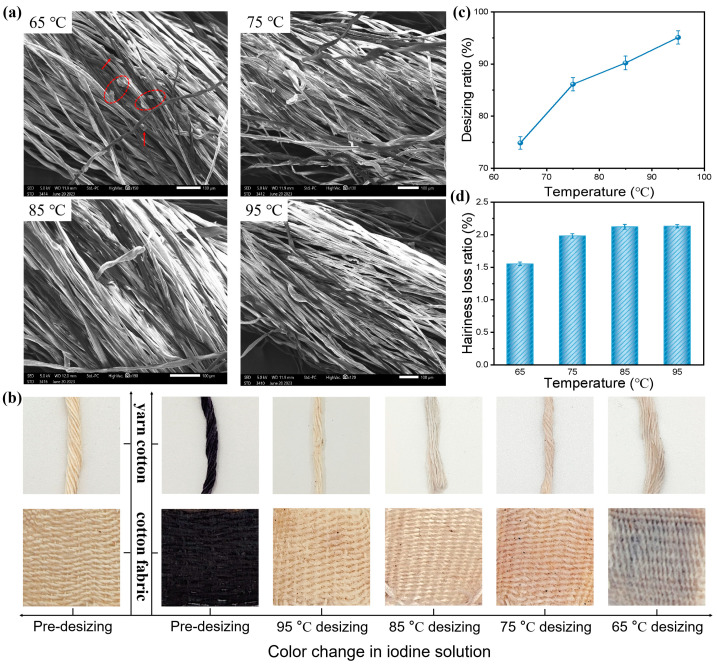
(**a**) SEM micrographs and (**b**) hairiness loss ratio after desizing at different temperatures. (**c**) Desizing ratio at different temperatures. (**d**) Color change in iodine solution before and after desizing.

In the textile process, the yarn frequently breaks, due to mechanical forces, so in the textile manufacturing process, warp sizing is used to improve the mechanical properties of the yarn, to better weave the fabric. However, prior to further dyeing procedures, it is imperative to eliminate the slurry adhered to the warp yarn in order to ensure optimal dyeing performance and fabric flexibility. Therefore, it is necessary to further evaluate the desizing performance of the sizing yarn. The desizing ratio, which measures the extent of desizing in relation to the upper sizing, is commonly employed as an indicator for evaluating the desizing ability of sizing yarns [36]. The optimal performance was observed with St-g-PAM 3 slurry, which has a starch to acrylamide mass ratio of 1:1.5, as discussed above. Consequently, we conducted further investigations on the desizing efficacy of the yarn treated with St-g-PAM 3 slurry. SEM images of the yarns after desizing the St-g-PAM 3 samples in water at different temperatures are shown in Figure 5a. It can be seen that the yarns were desized almost completely in water temperatures from 95 °C down to 75 °C, while there was a little residue of the slurry after desizing in water at 65 °C. Subsequently, we added 2–3 drops of a 10 g/L iodine aqueous solution onto the undesized yarn/fabric surface to facilitate a color reaction, thereby enabling a more visual assessment of any remaining starch content in the yarn. The undesized yarn/fabric exhibits a dark blue color upon encountering iodine, as depicted in Figure 5b. Subsequent desizing in water at 65 °C resulted in a lighter coloration, with only a faint shade of blue remaining, indicating the presence of residual modified starch on the surface of the yarn/fabric. This indicates that grafted starch can be desized in water temperatures in the 65–75 °C range, which reduces the energy consumption needed for high temperature desizing.

Furthermore, we conducted calculations on the desizing ratio of St-g-PAM 3 sizing in water at various temperatures, as illustrated in Figure 5c. The results indicate that the desizing ratio of the sizing increases proportionally with temperature elevation due to the degradation of the starch molecule structure and subsequent enhancement of its solubility, which is consistent with the results in Figure 3a above. Generally, in the desizing operation, more than 80% of starch should be removed from the sizing yarn, leaving residual sizing after desizing of less than 1% of the fabric weight, in order not to affect the subsequent process [37]. Therefore, when the desizing temperature is controlled at about 70 °C, it can meet the desizing requirements. In the desizing process, the heavy loss ratio of hairiness will cause a decrease in the mechanical properties of the yarn. Therefore, we evaluated the hairiness loss ratio of yarns desizing at different temperatures, as shown in Figure 5d. When the desizing temperature ranged from 65 °C to 95 °C, the yarn hairiness loss ratio was 1.55% (65 °C), 1.98% (75 °C), 2.12% (85 °C) and 2.13% (95 °C), respectively. It is obvious that the yarn hair loss ratio increases with the increase of the desizing temperature. However, as a whole, between the temperatures of 65 °C and 95 °C, the yarn hair loss ratio was relatively small, at less than 2.2%, which meets the requirements of yarn sizing [38].

Finally, we investigated the biochemical degradability by testing the BOD_5_/COD_cr_ values of the desizing wastewater of the St-g-PAM 3 prepared in this study and two commercially available PVA slurries. It was generally accepted that BOD_5_/COD_cr_ > 0.45 indicated excellent biochemical degradability, and BOD_5_/COD_cr_ < 0.25 indicated poor biochemical treatment. As can be seen from Table 1, the B/D values of the two PVA slurries were much less than 0.25, which indicated that they would be difficult to biochemically treat, while that of the grafted starch slurry had a B/D of = 0.476, which revealed an excellent biochemical degradability, and thus, St-g-PAM 3 has significant advantages and meets the requirements of the development of the modern textile industry. In terms of energy saving, the sizing process included: slurry boiling paste, pipeline slurry transfer, slurry tank soaking, slurry roller pressing (three cycles), drying, and manufacture by weaving. By adopting wet sizing technology, using squeezing rollers to control the yarn’s roll residue after dyeing and washing at about 60%, the yarn’s drying step before sizing is omitted. This changes the traditional sizing that needed to be carried out in the drying process before sizing, reducing by 30% the amount of steam in the process of drying before sizing, and therefore reducing the cost of production. The new technology is now the subject of a patent application. This method uses low temperatures (60 °C) for washing and desizing, requires no chemical additives, and saves energy consumption.

## 4. Conclusions

St-g-PAM pastes with different grafting percentages were prepared using different mass ratios of starch and monomer. The results showed that the graft copolymerization of starch with AM could overcome the drawbacks of starch such as brittleness in warp sizing and improve its end use properties. Basic characterization, such as infrared spectroscopy and EDS, confirmed that the successful introduction of PAM branches increased the hydrophilicity, anticoagulation and viscosity stability of starch, thus effectively improving the adhesion properties between starch and cotton fibers. The grafted PAM side chains shortened the rupture time of the pulp film in hot water and enhanced the tensile properties of the film as well as the abrasion resistance of the sizing yarn. These results indicate that the introduced PAM branched chains can reduce the brittleness of starch film and increase the toughness to protect the yarn and meet the weaving requirements. In addition, a comprehensive analysis of the performance of the slurry film and sizing yarns determined that St-g-PAM 3 slurry is optimal when the mass ratio of starch to acrylamide is 1:1.5, while maintaining a good desizing effect in hot water, and the above suggests that St-g-PAM slurry is expected to be used as a new type of environmentally friendly slurry for sizing cotton yarns.

## Figures and Tables

**Table 1:** BOD_5_ and COD_cr_ values of slurries.

	St-g-PAM	1788PVA	1799PVA
BOD_5_ (mg/L)	39,400	1630	800
COD_cr_ (mg/L)	82,773	181,000	182,000
BOD_5_/COD_cr_	0.476	0.009	0.004

## Data Availability

The data presented in this study are available upon request from the corresponding author.

## Supplementary Materials

The following supporting information can be downloaded at: https://www.mdpi.com/article/10.3390/polym16020182/s1, Figure S1: The Synthesis process of grafted starch slurry, Figure S2: SEM images of St-g-PAM 1, St-g-PAM 2 and St-g-PAM 4, Figure S3: EDS spectrogram of St-g-PAM 1, St-g-PAM 2 and St-g-PAM 4, Figure S4: Swelling power of slurry ACS and St-g-PAM with different raw material ratios, Figure S5: Optical photographs of the appearance of the slurry films ACS and St-g-PAM with different raw material ratios, Figure S6: Tensile strain of slurry films ACS and St-g-PAM with different raw material ratios, Figure S7: SEM cross-section images of unsized and sizing yarn, Figure S8: Breaking strength and moisture regain of sizing yarns ACS and St-g-PAM with different raw material ratios. Table S1: Specific synthetic details of graft copolymers, Table S2: Water solubility of St-g-PAM with different ratios at different temperatures, Table S3: Swelling power of St-g-PAM with different ratios at different temperatures, Table S4: Basic properties of the slurry.
