# Supplementary material for: Development of a Novel Energy Saving and Environmentally Friendly Starch via a Graft Copolymerization Strategy for Efficient Warp Sizing and Easy Removal
Source: Polymers (Basel). 2024 Jan 8;16(2):182. doi: 10.3390/polym16020182 (PMC10820382; doi:10.3390/polym16020182)
Supplement: Supplementary file 1 [file polymers-16-00182-s001.zip › polymers-2661193-supplementary.pdf]

polymers

# Development of a Novel Energy Saving and Environmentally Friendly Starch via a Graft Copolymerization Strategy for Efficient Warp Sizing and Easy Removal

Yuhan Zhu, Fei Guo, Jing Li, Zhen Wang, Zihui Liang*, Changhai Yi*

*National Local Joint Laboratory for Advanced Textile Processing and Clean Production, State Key Laboratory of New Textile Materials and Advanced Processing Technologies, Wuhan Textile University, Wuhan 430073, China*

Email: zhliang@wtu.edu.cn; ych@wtu.edu.cn

## Characterization

### Determination of St-g-PAM slurry

The 2 g starch sample was uniformly dispersed in 98 ml of distilled water and stirred in a water bath at 95 °C for 30 min to form St-g-PAM slurry. And then, take 20 ml St-g-PAM slurry and place it in a centrifuge tube and centrifuge for 10 min at a speed of 3000 r/min. Subsequently, the supernatant was placed in a glass petri dish and dried at 105 °C until constant weight. The water solubility and swelling power were calculated according to Eqs. (1) and (2)[39].

$$\text{Solubility (\%)}=\frac{5\times W_1}{W_3}\times 100 \quad (1)$$

$$\text{Swelling power (g/g)}=\frac{W_2-(W_3-W_1)}{W_3-W_1} \quad (2)$$

In order to distinguish from the mass "m" in formula (1), (2), and (3) in the manuscript, we use "w" to represent the mass here. Specifically, W1, W2 and W3 are the masses of the supernatant after drying to constant weight, the centrifuged sediment and the starch, respectively.

The transmittance of starch paste was determined according to the literature[25]. 1 g of starch sample was dispersed in 99 ml of distilled water, stirred in a water bath at 95 °C for 1 h to make the slurry paste completely, then cooled to room temperature. The transmittance was determined using UV-101 spectrometer and recorded at 4h intervals for four consecutive times.

### Preparation and measurement of St-g-PAM slurry film

The slurry film was prepared according to the literature[40]. 6% concentration of slurry was configured, stirred in a water bath at 95°C for 30 min, and then poured onto a horizontally placed polyester sheet after it was completely pasted, scraped and coated uniformly, and then dried to form a film under constant temperature and humidity. The starch film was cut into 100 mm × 20 mm strips, and a horizontal line was drawn in the middle position. After equilibrating at 65% relative humidity and 20 °C for 24 h, a weight of 1g was applied to one end of the starch film and placed in

distilled water at 95 °C, so that the horizontal line was exactly flush with the water surface, and the time of the starch film breaking was recorded as the time of the starch film's water solubilization. The breaking strength and elongation at break of the strip pulp film (130 mm × 10 mm) were tested in YG028 Instron universal materials testing machine, and each specimen was measured 20 times to take the average value. Moisture retention was used to assess the hygroscopicity of the slurry film by weighing a certain weight of slurry film and placing it in an oven at 105 °C. It was allowed to cool and then weighed. It was then weighed after being placed at 65% relative humidity and 20 °C for 24 h. Each specimen was measured three times to take the average value, and the moisture regain rate was calculated according to Eq. (4).

$$\text{Moisture regain (\%)}=\frac{M_2-M_1}{M_1}\times 100 \qquad (4)$$

where $M_1$ and $M_2$ refer to the dry weight and the weight of the slurry film after moisture absorption, respectively.

**Determination of St-g-PAM sizing yarns**

This experiment adopts the "three dipping and three rolling" process for sizing cotton yarns. The breaking strength and elongation at break and hairiness number of sizing yarns were determined according to the GB/T 3916-2013 and FZ/T 01086-2020. The abrasion resistance of the sizing yarns was determined by using FFZ622 yarn abrasion resistance tester, and the reciprocating speed of the grinding roller was set at 60 times/min to record the number of reciprocating friction times when the sizing yarns were ground off. Each specimen was tested 30 to take the average value. The breaking strength was calculated according to Eq. (5).

$$\text{Breaking strength (N / }mm^{-2}\text{)}=P/(A\times B) \qquad (5)$$

where P, A and B refer to the average breaking strength, average thickness and width of the plasma film, respectively. where P, A and B refer to the average breaking force, average thickness and width of the film, respectively.

**Desizability**

The desizing rate of the sizing yarn was determined by hot water desizing

method. The desizing process is to cut small pieces of cotton cloth and place them in a beaker with hot water and rinse them for 1h at different temperatures (65 °C, 75 °C, 85 °C, 95 °C) with stirring and rinsing twice. Subsequently, iodine solution was added dropwise to the sample for testing, and after cleaning, it was placed in an oven to dry to constant weight, and weighed after cooling. The desizing ratio and hairiness loss ratio were calculated according to Eq. (6).

$$\text{Desizing ratio (\%)} = \frac{T_1 - T_2}{T_1} \times 100 \qquad (6)$$

Where $T_1$ and $T_2$ denote the dry weight of sizing and desizing yarns, respectively.

$$\text{Hairiness loss ratio (\%)} = \frac{S\text{-}S1}{S} \times 100 \qquad (7)$$

where S and S1 refer to the dry weight of the original and desizing yarns, respectively.

**Figure S1**

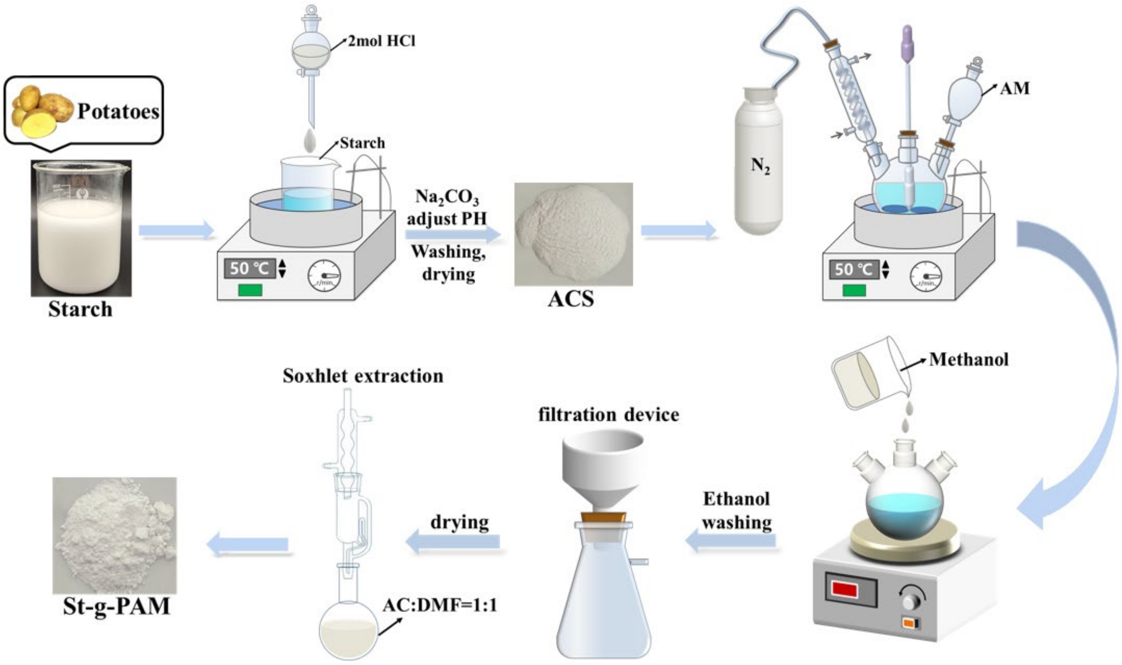


**Figure S1.** The Synthesis process of grafted starch slurry.

**Figure S2**

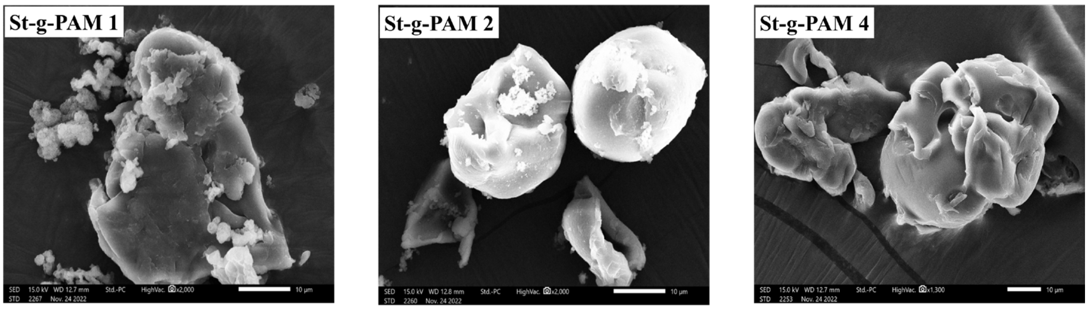


**Figure S2**. SEM images of St-g-PAM 1, St-g-PAM 2 and St-g-PAM 4.

## Figure S3

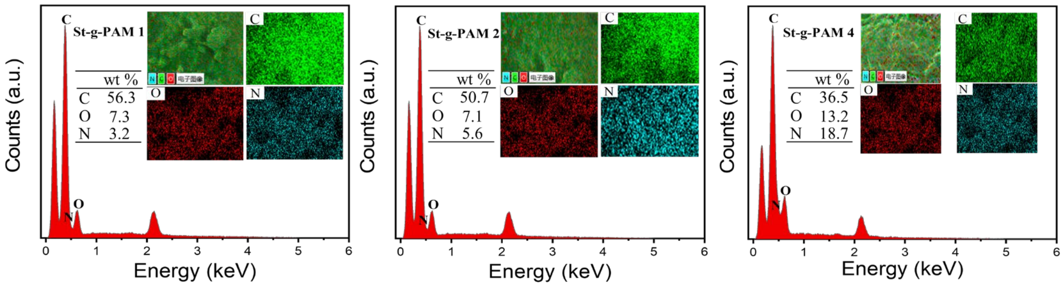


**Figure S3**. EDS spectrogram of St-g-PAM 1, St-g-PAM 2 and St-g-PAM 4.

**Figure S4**

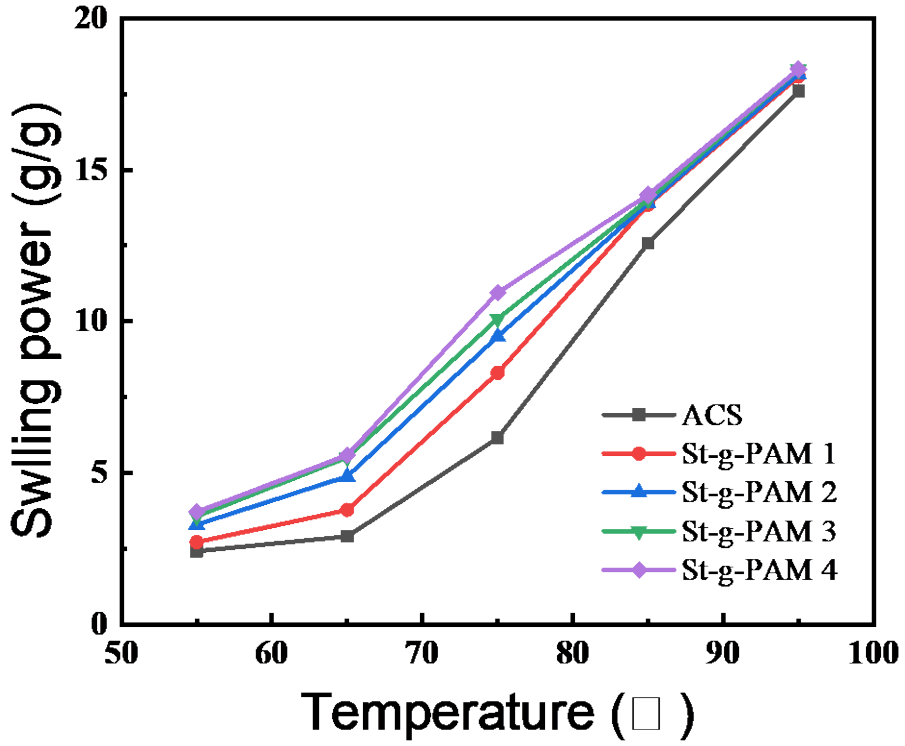


**Figure S4**. Swelling power of slurry ACS and St-g-PAM with different raw material ratios.

**Figure S5**

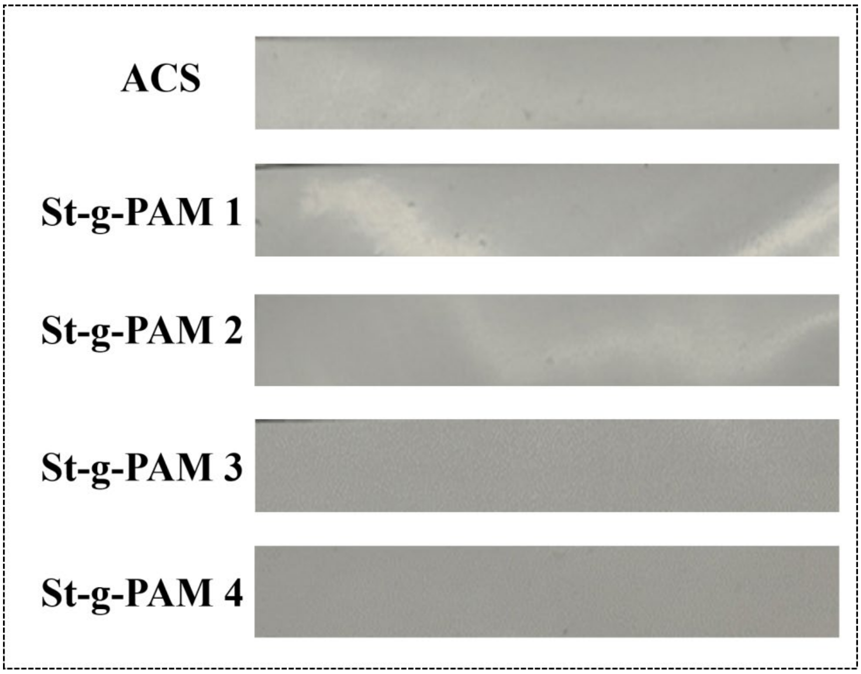


**Figure S5**. Optical photographs of the appearance of the slurry films ACS and St-g-PAM with different raw material ratios

**Figure S6**

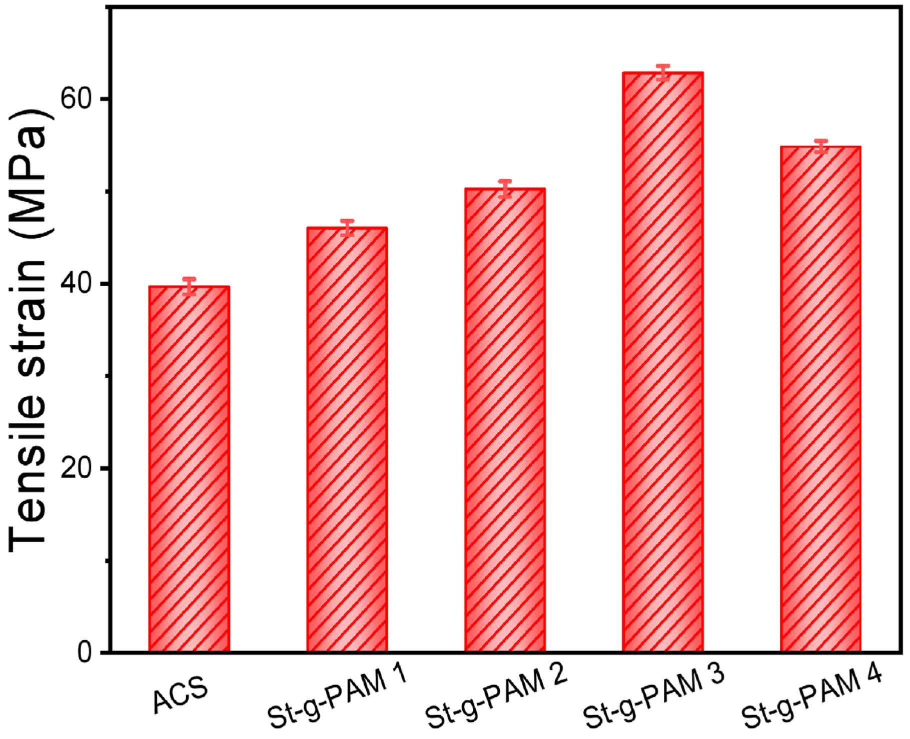


**Figure S6**. Tensile strain of slurry films ACS and St-g-PAM with different raw material ratios.

**Figure S7**

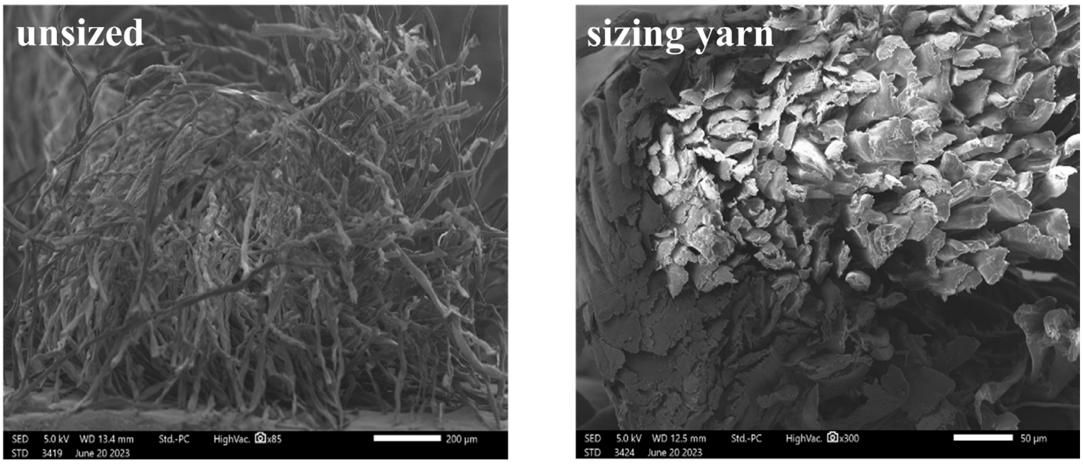


**Figure S7**. SEM cross-section images of unsized and sizing yarn.

**Figure S8**

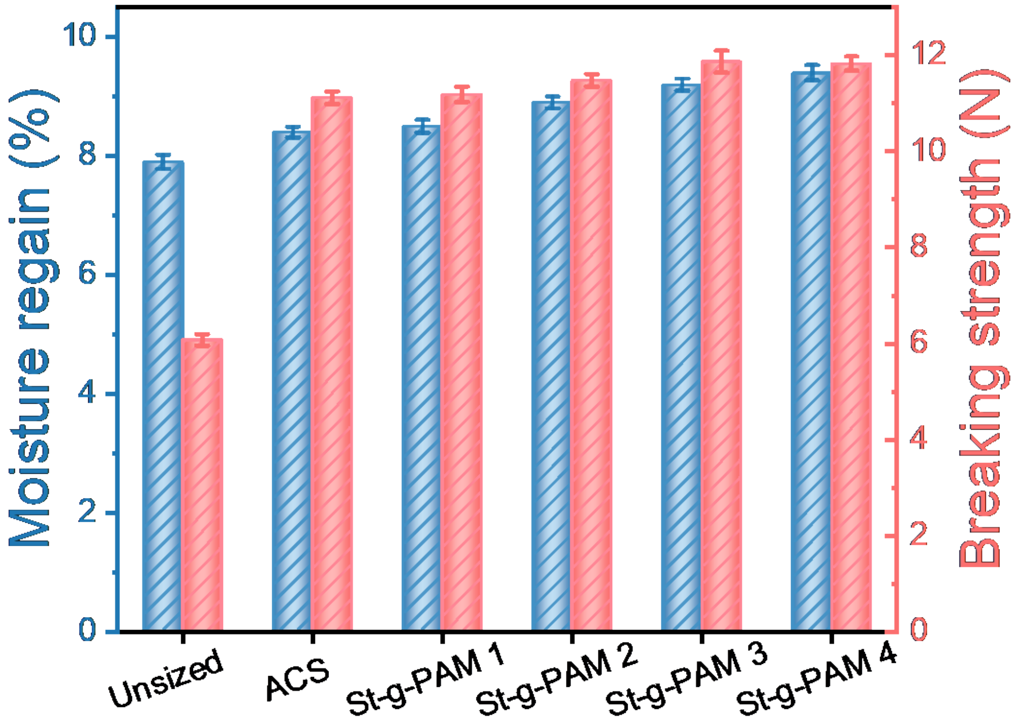


**Figure S8**. Breaking strength and moisture regain of sizing yarns ACS and St-g-PAM with different raw material ratios.

**Table S1**:

**Table S1**. Specific synthetic details of graft copolymers

| Grade | Starch (g) | AM (g) | Grafting ratio (%) | Graft efficiency (%) | Monomer conversion ratio (%) |
|---|---|---|---|---|---|
| Starch (St) | — | — | — | — | — |
| St-g-PAM 1 | 2 | 1 | 53.2 | 87.1 | 97.3 |
| St-g-PAM 2 | 1 | 1 | 55.8 | 86.5 | 97.9 |
| St-g-PAM 3 | 1 | 1.5 | 60.3 | 72.8 | 97.7 |
| St-g-PAM 4 | 1 | 2 | 64.6 | 58.9 | 97.3 |

**Table S2**:

**Table S2**. Water solubility (S) of St-g-PAM with different ratios at different temperatures

| Samples | 55 °C | | 65 °C | | 75 °C | | 85 °C | | 95 °C | |
|---|---|---|---|---|---|---|---|---|---|---|
| | $W_1$ (g) | S (%) | $W_1$ (g) | S (%) | $W_1$ (g) | S (%) | $W_1$ (g) | S (%) | $W_1$ (g) | S (%) |
| ACS | 0.0033 | 0.83 | 0.099 | 24.75 | 0.2246 | 56.15 | 0.2869 | 71.72 | 0.3196 | 79.23 |
| St-g-PAM 1 | 0.0411 | 10.28 | 0.102 | 25.51 | 0.2296 | 57.41 | 0.3068 | 76.71 | 0.3214 | 80.34 |
| St-g-PAM 2 | 0.1223 | 30.57 | 0.1923 | 48.07 | 0.2557 | 63.93 | 0.3036 | 75.89 | 0.3269 | 81.73 |
| St-g-PAM 3 | 0.1714 | 42.86 | 0.2208 | 55.21 | 0.2706 | 67.66 | 0.3039 | 75.98 | 0.3273 | 81.83 |
| St-g-PAM 4 | 0.1935 | 48.38 | 0.2388 | 59.77 | 0.2724 | 68.11 | 0.3042 | 76.05 | 0.3274 | 81.85 |

* $W_3$: the masses of the starch sample, 2g.

**Table S3:**

**Table S3**. Swelling power (Sp) of St-g-PAM with different ratios at different temperatures

| Samples | 55 ℃ | | 65 ℃ | | 75 ℃ | | 85 ℃ | | 95 ℃ | |
|---|---|---|---|---|---|---|---|---|---|---|
| | $W_2$ (g) | Sp (g/g) | $W_2$ (g) | Sp (g/g) | $W_2$ (g) | Sp (g/g) | $W_2$ (g) | Sp (g/g) | $W_2$ (g) | Sp (g/g) |
| ACS | 6.84 | 2.43 | 7.44 | 2.91 | 12.7 | 6.15 | 23.26 | 12.58 | 31.3 | 17.6 |
| St-g-PAM 1 | 7.28 | 2.72 | 9.08 | 3.78 | 16.46 | 8.3 | 25.14 | 13.85 | 32.03 | 18.08 |
| St-g-PAM 2 | 8.08 | 3.3 | 10.64 | 4.89 | 18.34 | 9.51 | 25.28 | 13.9 | 32.06 | 18.16 |
| St-g-PAM 3 | 8.36 | 3.57 | 11.58 | 5.51 | 19.66 | 10.09 | 25.48 | 14.02 | 32.32 | 18.32 |
| St-g-PAM 4 | 8.52 | 3.72 | 11.6 | 5.59 | 20.64 | 10.95 | 25.74 | 14.18 | 32.33 | 18.33 |

* $W_3$: the masses of the starch sample, 2g.

**Table S4**:

**Table S4.** Basic properties of the slurry

| Samples | Temperature (°C) | PH | Solidity (%) | | sizing ratio (%) |
|---|---|---|---|---|---|
| | | | setpoint | measured | |
| ACS | 94.9 | 6.73 | 6.0 | 5.14 | 8.32 |
| St-g-PAM 1 | 94.7 | 3.72 | 6.0 | 5.19 | 8.33 |
| St-g-PAM 2 | 94.8 | 3.95 | 6.0 | 5.18 | 8.43 |
| St-g-PAM 3 | 94.8 | 3.63 | 6.0 | 5.21 | 8.83 |
| St-g-PAM 4 | 94.9 | 3.74 | 6.0 | 5.25 | 9.48 |
